# Supplementary material for: Mapping eQTL by leveraging multiple tissues and DNA methylation
Source: BMC Bioinformatics. 2017 Oct 18;18:455. doi: 10.1186/s12859-017-1856-9 (PMC5648503; doi:10.1186/s12859-017-1856-9)
Supplement: Supplementary file 1 — Supplementary material. Supplementary material expanding on 1) Our model, 2) Individual components of our joint score test statistic, 3) Description of various null hypotheses, 4) Null and power simulations of our joint score test statistic, 5) Gibbs et al. dataset preprocessing, 6) Design of our data analysis, 7) KEGG pathway analysis on the results from Gibbs et al brain data, 8) JAGUAR, 9) A potential strategy to combine two models to maximize eQTL discovery, and 10) Reproducibility. (PDF 635 kb) [file 12859_2017_1856_MOESM1_ESM.pdf]

Supplementary material for “Mapping eQTL by leveraging multiple tissues and DNA methylation”

Chaitanya R. Acharya, Kouros Owzar and Andrew S. Allen

Contents

1 Our model **2**

2 Individual components of our joint score test statistic **2**

2.1 Additive genetic effect on the gene expression under the global null 2

2.2 The effect of SNP on gene expression via differential methylation patterns under the global null ($G \times M$ effect) 3

2.3 The tissue-specific effect due to genotype on the gene expression under the global null ($G \times T$ effect) 3

2.4 Latent effect (masking effect) of SNP on gene expression via tissue-specific methylation patterns ($G \times M \times T$ effect) 4

2.5 Joint score test statistic 4

3 Description of null hypotheses **5**

4 Evaluating our joint score test statistic **5**

5 Gibbs et al Data Preprocessing **7**

5.1 Genotype data 8

5.2 Gene Expression data 9

5.3 Methylation data 10

6 Data analysis design **11**

7 Results from applying KEGG pathway analysis on results from Gibbs et al data **12**

8 JAGUAR **13**

9 A potential strategy to combine two models to maximize eQTL discovery **14**

9.1 Testing the combined effect of methylation and genotype on multi-tissue eQTL detection 14

9.2 Testing the effect of genotype on multi-tissue eQTL detection 14

9.3 Omnibus test – a potential strategy 14

10 Reproducibility of the analysis **15**

1 Our model

For a given gene-SNP pair, gene expression is modeled as a function of genotype and methylation -

$$Y = J\alpha + G\beta + M\lambda + MG\phi + Au + Bv + Cw + Dx + \xi \tag{1}$$

where Y is nt-dimensional vector of expression levels in t tissues and n individuals, α is a vector of tissue-specific intercepts, G is nt-dimensional vector of genotypes, β is a fixed effect of genotype across tissue, M is nt-dimensional vector of methylation levels, λ is an overall methylation-specific fixed effect, MG is nt-dimensional vector of the product of methylation and genotype, ϕ is the regression coefficient for genotype and methylation interaction (fixed effect), $u \sim N\left(0, \tau AA^T\right)$ is a vector of subject-specific random effect, $v \sim N\left(0, \gamma BB^T\right)$ is a vector of tissue-specific random effects, $w \sim N\left(0, \delta CC^T\right)$ is a vector of tissue-specific random effects that describes the interaction effect between genotype, methylation is a vector of random effects describing the interaction between genotype, methylation and tissue, $x \sim N\left(0, \theta DD^T\right)$ is a vector of tissue-specific random effects describing red tissue-specific methylation effects and $\xi \sim N\left(0, \epsilon I_{nt}\right)$. The matrices J, A, B, C, and D are design matrices with B being a function of genotype, C is a function of both genotype and methylation data and finally, D is a function of just the methylation data. J is $nt \times t$ dimensional matrix denoting the design matrix for the tissue-specific intercepts. A is $nt \times n$ design matrix for the subject-specific intercepts. B is a $nt \times t$ design matrix of stacked genotypes. C is a $nt \times t$ design matrix of stacked (product of) tissue-specific methylation and genotype data. D is $nt \times t$ design matrices of stacked tissue-specific methylation data. The parameters of interest are γ, δ, β and ϕ; α, λ, τ, θ and ϵ are nuisance parameters.

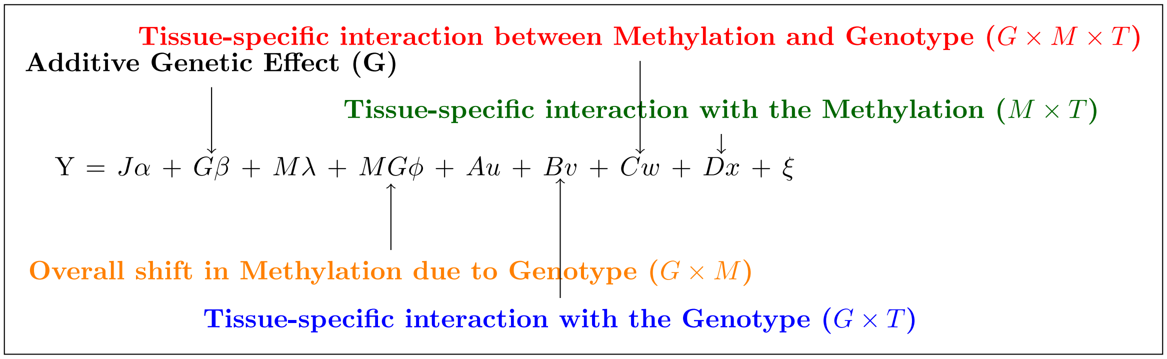

Figure S1: Description of all the terms in our model

Parameters of interest are γ, δ, β and ϕ; α, λ, τ, θ and ϵ are nuisance parameters. We test the null hypothesis that $H_0 : \beta = \phi = \gamma = \delta = 0$, i.e. the variant does not affect gene expression across any of the tissues. Our joint score test will test for the effect of genotype on 1) an overall shift in the gene expression, 2) tissue-specific interaction ($G \times T$), 3) overall methylation ($G \times M$), and 4) tissue-specific methylation ($G \times M \times T$)

2 Individual components of our joint score test statistic

2.1 Additive genetic effect on the gene expression under the global null

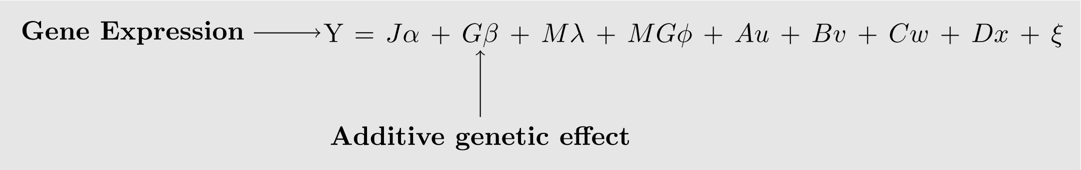

The score test for the fixed effect β takes the following form under the global null –

$$U_\beta = \left(G - \bar{G}\right)^T \Sigma_n^{-1} \hat{Y} \tag{2}$$

where $\left(G - \bar{G}\right)$ is a vector of mean-centered genotypes for all individuals and $\hat{Y} = \left(Y - J\hat{\alpha} - M\hat{\lambda}\right)$. U_β is a scalar quantity in a linear form and follows a χ_1^2 distribution.

Squaring U_β gives us the following quadratic form, which will be useful while aggregating all the score test statistics.

$$U_\beta^2 = \hat{Y}^T \Sigma_n^{-1} \left(G - \bar{G}\right) \left(G - \bar{G}\right)^T \Sigma_n^{-1} \hat{Y} \tag{3}$$

2.2 The effect of SNP on gene expression via differential methylation patterns under the global null ($G \times M$ effect)

Gene Expression $\longrightarrow$ $Y = J\alpha + G\beta + M\lambda + MG\phi + Au + Bv + Cw + Dx + \xi$

$\uparrow$ **Interaction effect between methylation and genotype ($G \times M$)**

The score test for the interaction effect ϕ takes the following form under the global null –

$$U_\phi = \left(MG - \overline{MG}\right)^T \Sigma_n^{-1} \hat{Y} \tag{4}$$

where $\Sigma_n = diag\left(\Sigma, \cdots, \Sigma\right)$ is an $nt \times nt$ block diagonal matrix and $\hat{Y} = \left(Y - J\hat{\alpha} - M\hat{\lambda}\right)$. U_ϕ is a scalar quantity in a linear form and follows a χ_1^2 distribution. Squaring U_ϕ gives us the following quadratic form, which will be useful while aggregating all the score test statistics.

$$U_\phi^2 = \hat{Y}^T \Sigma_n^{-1} \left(MG - \overline{MG}\right) \left(MG - \overline{MG}\right)^T \Sigma_n^{-1} \hat{Y} \tag{5}$$

2.3 The tissue-specific effect due to genotype on the gene expression under the global null ($G \times T$ effect)

Gene Expression $\longrightarrow$ $Y = J\alpha + G\beta + M\lambda + MG\phi + Au + Bv + Cw + Dx + \xi$

$\uparrow$ **Interaction effect between genotype and tissues ($G \times T$)**

The score for the variance component $\boldsymbol{\gamma}$ under the global null is –

$$\frac{1}{2}\left\{\hat{Y}^T \Sigma_n^{-1} B B^T \Sigma_n^{-1} \hat{Y} - Tr\left(\Sigma_n^{-1} B B^T\right)\right\} \tag{6}$$

where $\Sigma_n = diag\left(\Sigma, \cdots, \Sigma\right)$ is an $nt \times nt$ block diagonal matrix and $\hat{Y} = \left(Y - J\hat{\alpha} - M\hat{\lambda}\right)$. As the *trace* term does not depend on the data, we use the first term to construct the test statistic.

$$U_\gamma = \frac{1}{2}\hat{Y}^T\Sigma_n^{-1}BB^T\Sigma_n^{-1}\hat{Y} \tag{7}$$

U_γ follows a mixture of chi-square distribution and the p value is approximated using a scaled χ^2 distribution (the Satterthwaite method) by matching the first two moments as $U_\gamma \sim \kappa\chi^2_\nu$ where $\kappa = \frac{Var(U_\gamma)}{2E[U_\gamma]}$ and $\nu = \frac{2E[U_\gamma]^2}{Var(U_\gamma)}$.

2.4 Latent effect (masking effect) of SNP on gene expression via tissue-specific methylation patterns ($G \times M \times T$ effect)

Gene Expression $\longrightarrow Y = J\alpha + G\beta + M\lambda + MG\phi + Au + Bv + Cw + Dx + \xi$

$\uparrow$ (pointing to Cw)

Three-way interaction between methylation, genotype and tissue ($G \times M \times T$)

The score for the variance component $\boldsymbol{\delta}$ under the global null is –

$$\frac{1}{2}\left\{\hat{Y}^T\Sigma_n^{-1}CC^T\Sigma_n^{-1}\hat{Y} - Tr\left(\Sigma_n^{-1}CC^T\right)\right\} \tag{8}$$

where $\Sigma_n = diag\left(\Sigma, \cdots, \Sigma\right)$ is an $nt \times nt$ block diagonal matrix and $\hat{Y} = \left(Y - J\hat{\alpha} - M\hat{\lambda}\right)$. As the *trace* term does not depend on the data, we use the first term to construct the test statistic.

$$U_\delta = \frac{1}{2}\hat{Y}^T\Sigma_n^{-1}CC^T\Sigma_n^{-1}\hat{Y} \tag{9}$$

U_δ follows a mixture of chi-square distribution, the p value can be approximated using a scaled χ^2 distribution by matching the first two moments as $U_\delta \sim \kappa\chi^2_\nu$ where $\kappa = \frac{Var(U_\delta)}{2E[U_\delta]}$ and $\nu = \frac{2E[U_\delta]^2}{Var(U_\delta)}$.

2.5 Joint score test statistic

We propose a weighted sum of the above components to arrive at our joint score test statistic, U_ζ. Since U_β and U_ϕ are linear in Y while U_γ and U_δ are quadratic, we propose the following rule to combine them –

$$\begin{aligned} U_\zeta &\equiv \left(\boldsymbol{a_\beta}U_\beta^2 + \boldsymbol{a_\phi}U_\phi^2 + \boldsymbol{a_\gamma}U_\gamma + \boldsymbol{a_\delta}U_\delta\right) \\ &\equiv \hat{Y}^T\hat{\Sigma}_n^{-1}\left[a_\beta\left(G - \bar{G}\right)\left(G - \bar{G}\right)^T + a_\phi\left(MG - \overline{MG}\right)\left(MG - \overline{MG}\right)^T + a_\gamma\frac{1}{2}BB^T + a_\delta\frac{1}{2}CC^T\right]\hat{\Sigma}_n^{-1}\hat{Y} \end{aligned} \tag{10}$$

where a_β, a_ϕ, a_γ and a_δ are scalar constants chosen to minimize the variance of U_ψ. Under the null, U_ψ is distributed as a mixture of chi-square random variables. We use Satterthwaite method [1] to approximate the p values from a scaled χ^2 distribution by matching the first two moments as $U_\psi \sim \kappa\chi^2_\nu$ where $\kappa = \frac{Var(U_\psi)}{2E[U_\psi]}$ and $\nu = \frac{2E[U_\psi]^2}{Var(U_\psi)}$.

Our joint score test will test for the effect of genotype on 1) an overall shift in the gene expression, 2) tissue-specific interaction ($G \times T$), 3) overall methylation ($G \times M$), and 4) tissue-specific methylation ($G \times M \times T$)

3 Description of null hypotheses

Since we are testing multiple models and comparing the results, it is important to consider the null hypotheses being tested. Even though, the overarching hypothesis is to test whether a SNP is associated with a gene, more technically, each model is testing a specific hypothesis. For example, a tissue-by-tissue (TBT) method applies the following model

$$Y = G\beta + \xi \qquad \xi \sim N(0, \epsilon)$$

where Y is the gene expression and G is the genotype. We test whether $\underline{H_0 : \beta = 0}$ across all the tissues.

Similarly, JAGUAR, which applies the following model

$$Y = J\alpha + G\beta + Au + Bv + \xi \qquad \xi \sim N(0, \epsilon)$$

where Y is the gene expression data, J is the tissue-specific intercept, G is the genotype, A forms the design matrix for the individual specific random effects, B forms the design matrix for the genotype × tissue. $u \sim N(0, \tau)$ and $v \sim N(0, \gamma)$. We test whether $\underline{H_0 : \beta = \gamma = 0}$.

We have already discussed the null hypothesis of our model in the previous section.

Method	Model	Null Hypothesis
TBT-eQTL	$Y = G\beta + \epsilon$	$\beta = 0$
JAGUAR	$Y = J\alpha + G\beta + Au + Bv + \xi$	$\beta = \gamma = 0$
Our method	$Y = J\alpha + G\beta + M\lambda + MG\phi + Au + Bv + Cw + Dx + \xi$	$\beta = \phi = \gamma = \delta = 0$

4 Evaluating our joint score test statistic

For a positive integer t that represents number of tissues, if $\mathbf{1}$ denotes a column vector of t ones and $\mathbb{I}$ denotes the corresponding $t \times t$ diagonal matrix, following the t-variate normal law denoted by $N_t[\mu, \Sigma]$ with mean $\mu \in \mathbb{R}^t$ and variance $\Sigma \in \mathbb{R}^{t\times t}$, expression levels of a target gene j at a single locus by using the following vectorized form of the linear mixed model –

$$y_{ij} = \alpha_j + \mathbf{1}\beta_j g_i + \mathbf{1}\lambda_j m_{ij} + \mathbf{1}\phi_j m_{ij} g_i + \mathbf{1}a_i + b_j g_i + c_j m_{ij} g_i + d_j m_{ij} + \xi_{ij} \qquad \xi_{ij} \overset{i.i.d.}{\sim} N(0, \epsilon\mathbb{I}) \tag{11}$$

where y_{ij} is a $t\times 1$ vector of gene expression data, α_t is the tissue-specific intercept ($\alpha_t \in \mathbb{R}^t$), β_j describes the main additive genotypic effect ($\beta_j \in \mathbb{R}^1$), λ_j describes the overall effect due to methylation ($\lambda_j \in \mathbb{R}^1$), ϕ describes the interaction effect between the overall methylation and genotype ($\phi_j \in \mathbb{R}^1$), g_i is the value of a bi-allelic genotype such that $g \in (0, 1, 2)$ represents the number of copies of the minor allele. The random effect $b_j \in \mathbb{R}^t$ represents tissue-specific effect of the genotype, $c_j \in \mathbb{R}^t$ represents tissue-specific interaction effect between methylation and genotype, $d_j \in \mathbb{R}^t$ represents tissue-specific methylation effect, and $a_i \in \mathbb{R}^1$ is a subject-specific random intercept. We assume that all the random effects are independent and that $a_i \sim N_1(0, \tau)$, $b_j \sim N_t(0, \gamma\mathbb{I})$, $c_j \sim N_t(0, \delta\mathbb{I})$ and $d_j \sim N_t(0, \theta\mathbb{I})$. Methylation data for 5 tissues was generated independently from a multivariate normal distribution with mean zero and positive definite variance-covariance matrix.

Under the global null, the reduced model is –

$$y_{ij} = \alpha_j + \mathbf{1}\lambda_j m_{ij} + \mathbf{1}a_i + d_j m_{ij} + \xi_{ij} \qquad \xi_{ij} \overset{i.i.d.}{\sim} N(0, \epsilon\mathbb{I}) \tag{12}$$

We use 1,000 data replicates to evaluate type I error and power calculations. Simulations were performed by varying β, the proportion of variance explained by the random effect describing the interaction between genotype and tissue, $G \times T = PVE_\gamma \equiv \left(\frac{\gamma}{\theta+\tau+\epsilon+\gamma+\delta}\right)$, and the proportion of variance explained by the random effect describing the interaction between genotype, methylation and tissue, $G \times M \times T = PVE_\delta \equiv \left(\frac{\delta}{\theta+\tau+\epsilon+\gamma+\delta}\right)$.

A linear mixed effects model was fit using the package *lme4* [2] in the statistical environment R (R Core Team) [3].

We evaluate our score test statistic and its individual components by starting with 100 samples and 5 tissues.

G Effect	$G \times M$ Effect	$PVE_{G\times M\times T}$	$PVE_{G\times T}$	$U^2_{\beta\|H_0}$	$U^2_{\phi\|H_0}$	$U_{\gamma\|H_0}$	$U_{\delta\|H_0}$	$U_{\psi\|H_0}$
NO	NO	0	0	0.058	0.045	0.061	0.053	0.06
NO	NO	0	5	0.071	0.055	0.278	0.052	0.161
NO	NO	0	8	0.092	0.064	0.602	0.062	0.427
NO	NO	5	0	0.053	0.151	0.047	0.28	0.173
NO	NO	5	5	0.079	0.153	0.294	0.288	0.325
NO	NO	5	8	0.107	0.143	0.641	0.274	0.571
NO	NO	8	0	0.055	0.251	0.072	0.549	0.383
NO	NO	8	5	0.081	0.255	0.312	0.622	0.585
NO	NO	8	8	0.107	0.263	0.645	0.604	0.734
NO	YES	0	0	0.058	0.883	0.039	0.674	0.171
NO	YES	0	5	0.08	0.884	0.28	0.696	0.385
NO	YES	0	8	0.101	0.888	0.629	0.674	0.616
NO	YES	5	0	0.047	0.825	0.061	0.772	0.381
NO	YES	5	5	0.065	0.844	0.314	0.751	0.525
NO	YES	5	8	0.102	0.834	0.611	0.747	0.725
NO	YES	8	0	0.071	0.762	0.072	0.83	0.573
NO	YES	8	5	0.084	0.76	0.309	0.837	0.677
NO	YES	8	8	0.099	0.719	0.579	0.848	0.826
YES	NO	0	0	0.287	0.05	0.054	0.045	0.208
YES	NO	0	5	0.308	0.058	0.295	0.055	0.357
YES	NO	0	8	0.322	0.059	0.648	0.056	0.588
YES	NO	5	0	0.303	0.127	0.053	0.275	0.355
YES	NO	5	5	0.301	0.147	0.291	0.253	0.484
YES	NO	5	8	0.356	0.116	0.642	0.249	0.689
YES	NO	8	0	0.325	0.279	0.064	0.566	0.585
YES	NO	8	5	0.323	0.268	0.306	0.584	0.68
YES	NO	8	8	0.329	0.284	0.631	0.606	0.823
YES	YES	0	0	0.322	0.916	0.062	0.693	0.421
YES	YES	0	5	0.327	0.88	0.308	0.669	0.552
YES	YES	0	8	0.341	0.89	0.637	0.691	0.74
YES	YES	5	0	0.318	0.81	0.084	0.742	0.57
YES	YES	5	5	0.305	0.809	0.294	0.734	0.666
YES	YES	5	8	0.349	0.802	0.589	0.757	0.809
YES	YES	8	0	0.288	0.761	0.076	0.832	0.705
YES	YES	8	5	0.32	0.767	0.333	0.84	0.816
YES	YES	8	8	0.351	0.737	0.623	0.817	0.869

Table S1: Table comparing the statistical power of the joint score test statistic, U_ψ and the contributions from its main components, U^2_β, U^2_ϕ, U_γ and U_δ, all under the global null. These data were generated from 1,000 simulations run on 500 individuals and five tissues with genotypes generated at a common variant allele frequency (MAF = 0.3).

We change the number of tissues from 5 to 10 to investigate how varying tissue numbers affect the statistical power of our model and its components.

G Effect	$G \times M$ Effect	$PVE_{G \times M \times T}$	$PVE_{G \times T}$	$U^2_{\beta\|H_0}$	$U^2_{\phi\|H_0}$	$U_{\gamma\|H_0}$	$U_{\delta\|H_0}$	$U_{\psi\|H_0}$
NO	NO	0	0	0.035	0.052	0.045	0.048	0.044
NO	NO	0	5	0.067	0.045	0.278	0.05	0.161
NO	NO	0	8	0.103	0.048	0.611	0.059	0.433
NO	NO	5	0	0.065	0.157	0.059	0.269	0.151
NO	NO	5	5	0.074	0.135	0.302	0.286	0.332
NO	NO	5	8	0.115	0.151	0.603	0.263	0.559
NO	NO	8	0	0.043	0.258	0.058	0.616	0.412
NO	NO	8	5	0.068	0.266	0.294	0.569	0.523
NO	NO	8	8	0.09	0.263	0.605	0.592	0.735
NO	YES	0	0	0.051	1	0.071	1	0.856
NO	YES	0	5	0.081	1	0.308	0.999	0.901
NO	YES	0	8	0.127	1	0.628	0.998	0.954
NO	YES	5	0	0.061	0.998	0.087	0.996	0.866
NO	YES	5	5	0.075	1	0.304	0.993	0.899
NO	YES	5	8	0.094	1	0.581	0.998	0.953
NO	YES	8	0	0.052	0.988	0.092	0.99	0.899
NO	YES	8	5	0.079	0.993	0.318	0.992	0.937
NO	YES	8	8	0.116	0.991	0.611	0.99	0.961
YES	NO	0	0	0.823	0.062	0.081	0.048	0.727
YES	NO	0	5	0.799	0.055	0.378	0.052	0.785
YES	NO	0	8	0.797	0.04	0.675	0.063	0.862
YES	NO	5	0	0.826	0.138	0.099	0.294	0.814
YES	NO	5	5	0.794	0.12	0.384	0.268	0.847
YES	NO	5	8	0.793	0.13	0.662	0.263	0.905
YES	NO	8	0	0.815	0.267	0.102	0.618	0.882
YES	NO	8	5	0.816	0.254	0.377	0.585	0.906
YES	NO	8	8	0.767	0.263	0.632	0.555	0.918
YES	YES	0	0	0.816	1	0.146	1	0.984
YES	YES	0	5	0.807	1	0.356	0.999	0.996
YES	YES	0	8	0.796	1	0.663	1	0.993
YES	YES	5	0	0.814	0.999	0.135	0.994	0.985
YES	YES	5	5	0.812	0.998	0.395	0.995	0.992
YES	YES	5	8	0.78	1	0.655	0.996	0.992
YES	YES	8	0	0.832	0.99	0.147	0.997	0.986
YES	YES	8	5	0.781	0.992	0.39	0.992	0.994
YES	YES	8	8	0.803	0.992	0.645	0.992	0.993

Table S2: Table comparing the statistical power of the joint score test statistic, U_ψ and the contributions from its main components, U^2_β, U^2_ϕ, U_γ and U_δ, all under the global null. These data were generated from 1,000 simulations run on 500 individuals and 10 tissues with genotypes generated at a common variant allele frequency (MAF = 0.3).

In an effort to evaluate the performance of our joint score test when compared with TBT-eQTL and JAGUAR in the presence and absence of methylation effects, power simulations on 1,000 data replicates in 500 samples across 5 and 10 tissues. We presented our results from one such simulation study (500 samples and 5 tissues, in the absence and presence of methylation effects) as Figures 2a and 2b in our main manuscript. Figure S2 in our supplementary section show results from running simulations in 500 samples and 10 tissues.

5 Gibbs et al Data Preprocessing

The following preprocessing is done on Gibbs *et al* data [4] comprising of 150 samples from four regions of normal brain (cerebellum, frontal cortex, pons and temporal cortex).

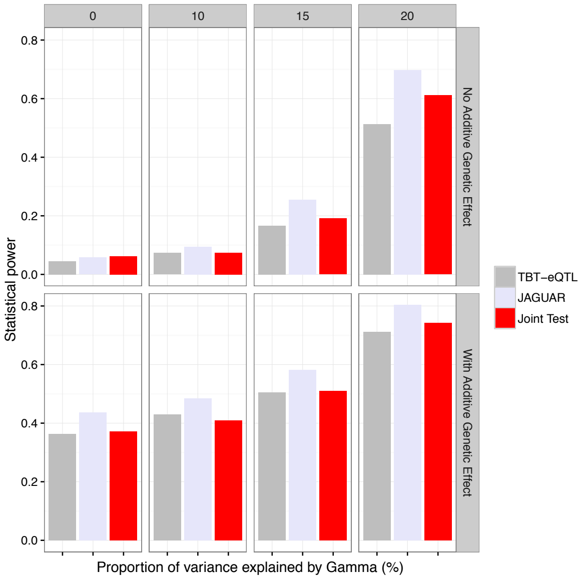

(a) In the absence of methylation effect

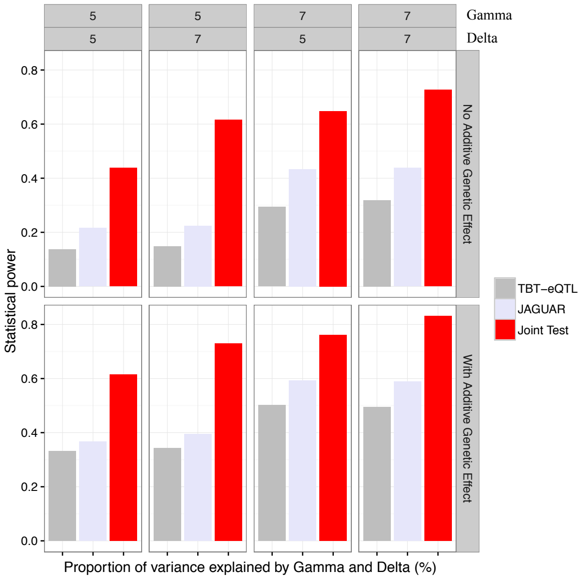

(b) In the presence of methylation effect

Figure S2: Mapping eQTLs in multiple tissues. Barplot depicting the statistical power comparison between TBT-eQTL, JAGUAR and our new (Joint Score Test) method. Left panel illustrates eQTL mapping in the absence of higher order methylation effects. Right panel illustrates eQTL mapping in the presence of higher order methylation effects and the numbers in the top two rows indicate the proportions of variance explained by both γ and δ, respectively. 1000 replicates across 500 samples and 10 tissues were used for power calculations.

Accession ID	Repository	Data type	Platform	Number of probes
GSE15745	GEO	Gene expression data	Illumina humanRef-8 v2.0 expression bead-chip	22,184
GSE15745	GEO	Methylation data	Illumina Human-Methylation27 BeadChip	27,578
phs000249.v1.p1	dbGaP	Genotype data	HumanHap550v3.0 7	561,466

Table S3: *A description of brain data*

5.1 Genotype data

The genotype data is recoded into a SNP matrix of values 0, 1 and 2 representing minor allele counts. Samples with African and Asian ancestry were removed from the analysis. These SNPs were filtered on the missing-ness of the individual data and the SNP data (excluded SNPs with missing data), followed by MAF (included SNPs with MAF $\geq$ 0.05)and Hardy-Weinberg equilibrium (HWE; p-values $\leq$ 0.001) in the same order using PLINK [5] software. SNPs with missing values were removed from the analysis. We ended with 400,097 SNPs after preprocessing.

5.2 Gene Expression data

Gene expression on four brain regions are publicly available as rank-invariant [6] normalized gene expression data ("series matrix file"). All the negative values in the gene expression dataset are changed to a 1 and the entire dataset was then log2 transformed. Before generating the PCA plots, samples with African and Asian ancestry (n = 2) were removed from the analysis in order to keep the study a homogenous mixture of European-Caucasians. All the gene expression probes on sex chromosomes X and Y were removed from the analysis.

Each gene expression probe was then adjusted for known variation contributed by batch effects and biological covariates such as tissue bank, gender, hybridization batch and numeric covariates such as post-mortem interval (PMI) and age as well as unknown variation using surrogate variable analysis (SVA) model [7].

$$\text{Gene Expression} = \text{Biological Covariates} + \text{Known Batch Effects} + \text{Unkown Variation} + \text{Measurement Error}$$

It was shown in the past that the number of *cis*-eQTL detected significantly improved when multiple PCs were removed from the expression data [8].

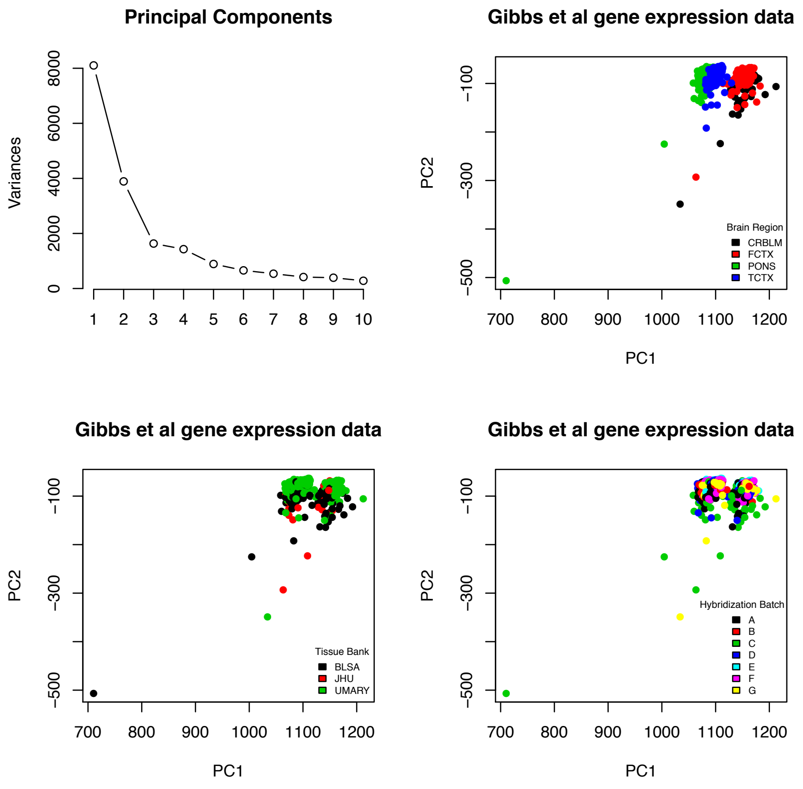

Figure S3: PCA plots exploring the presence of any biological or methodological variation using the first two principal components of the unadjusted rank-invariant normalized gene expression data.

5.3 Methylation data

Methylation data, obtained as a "series matrix file" consisted of Beta-values, which represent the ratio of methylated probe intensity and the overall intensity (sum of methylated and unmethylated probe intensities) [9]. We followed the previously mentioned method to preprocess methylation data using the SVA model. The biological covariates here include tissue bank, gender, hybridization batch and numeric covariates such as post-mortem interval (PMI) and age.

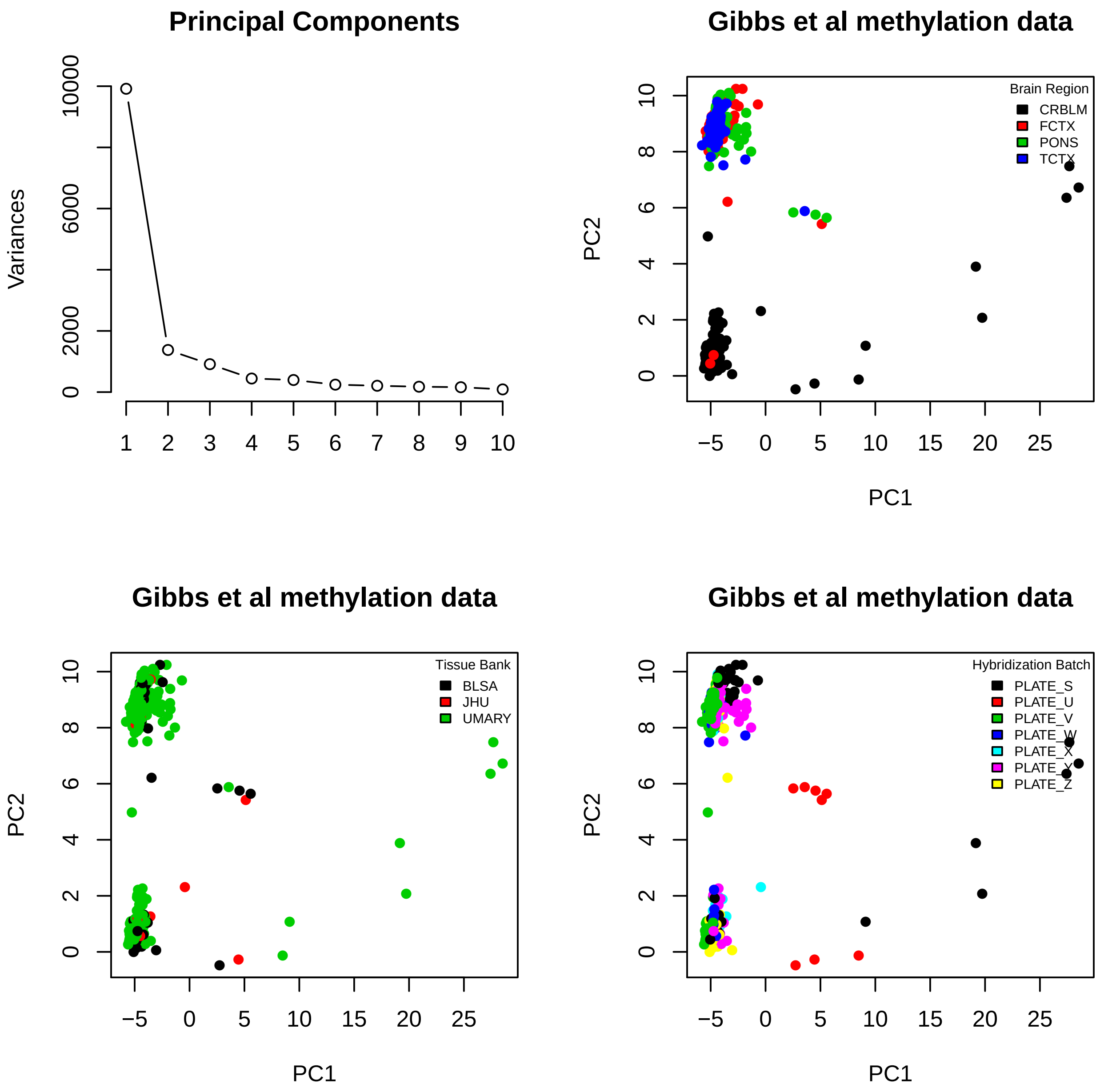

Figure S4: PCA plots exploring the presence of any biological or methodological variation using the first two principal components of the unadjusted methylation data.

6 Data analysis design

We performed data analyses that focused on *cis* candidate regions.

- The proximity of an eQTL to the transcription start site of a gene does not exceed 100 kilobase up- and down-stream of the transcription start site of a gene (*cis*-SNP).
- We picked CpG islands that are less than 1.5 kilobase up- and down-stream of the transcription start site of the same gene.

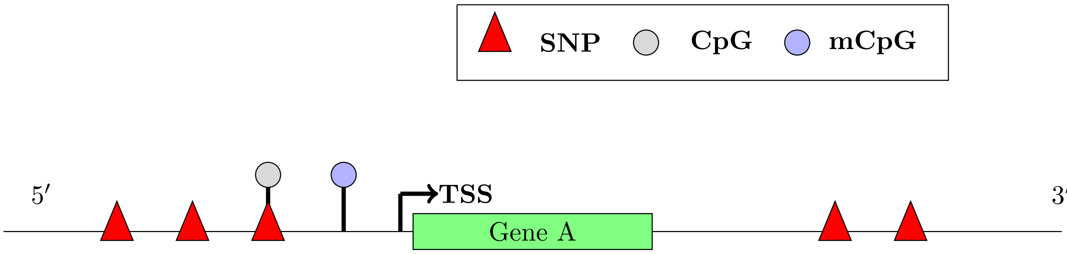

Figure S5: An illustration of the analysis design. The red triangles indicate SNPs and the circles, CpG sites (gray = unmethylated; blue = methylated). CpG sites that are at most 1.5 Kb from the transcription start site (TSS) of a gene were picked for the analysis. All the SNPs that were picked did not exceed 100 kilobase up- and down-stream of the transcription start site of a gene (*cis*-SNPs).

Each mRNA - CpG pair will be tested for a strong association with every *cis*-SNP. A total of 11,076 mRNA transcripts, 14,244 CpG sites and 144,393 *cis*SNPs were tested in our analysis. Below are the histogram plots that show their distribution across the genome.

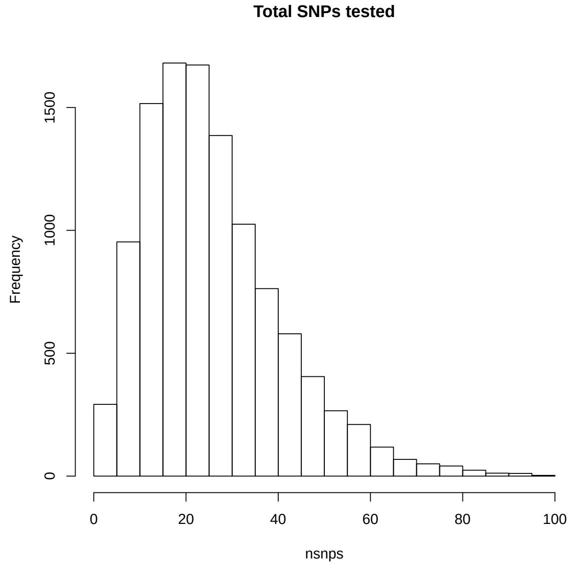

(a) Distribution of SNPs

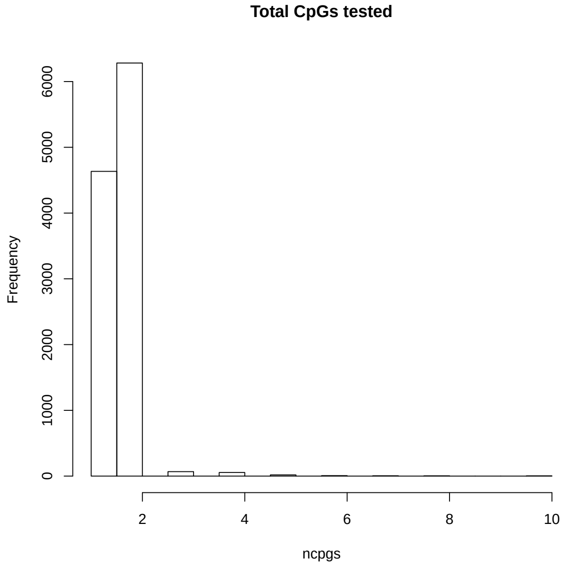

(b) Distribution of CpGs

Figure S6: Histogram of distribution of SNPs and CpGs tested in the genome. Most of the genes have 2 CpG sites in their promoter region.

Here's a qq-plot of the score test p values

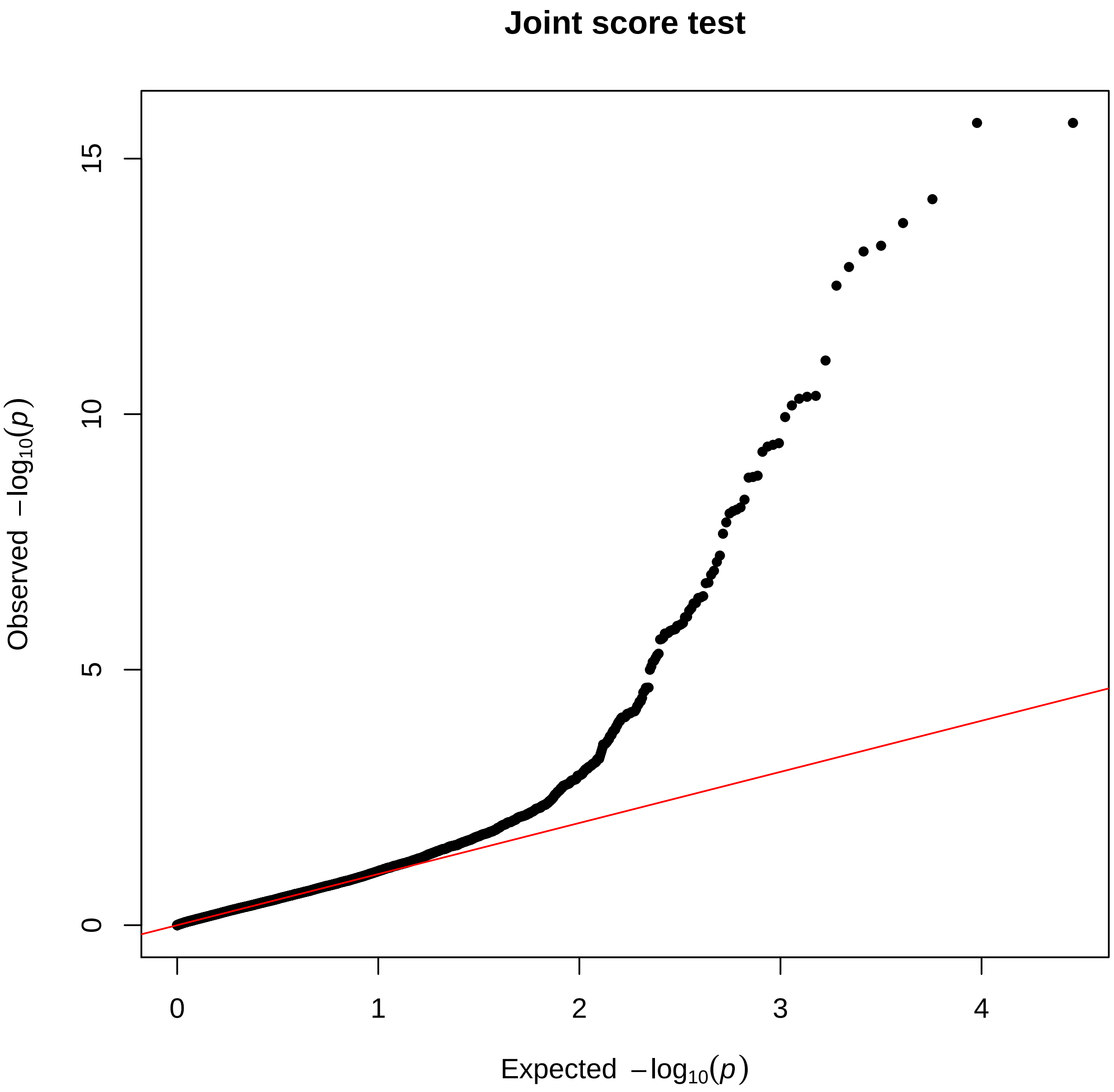

Figure S7: QQ-plot of the unadjusted p values obtained from our joint model.

7 Results from applying KEGG pathway analysis on results from Gibbs et al data

KEGG pathway analysis shows the biological relevance of this discovery.

An advantage to using our approach is the knowledge of individual contributions of each different effect, which gives us a hint on the extent to which tissue-specific effect is driving the association between every mRNA - CpG pair and SNP.

Cluster	KEGG ID	Pathway Name	qvalue
TBT	hsa01100	Metabolic pathways	0.019519816
JAGUAR	hsa00480	Glutathione metabolism	0.008110498
JAGUAR	hsa01100	Metabolic pathways	0.018006598
Joint score test	hsa01100	Metabolic pathways	0.00014368
Joint score test	hsa03010	Ribosome	0.00014368
Joint score test	hsa00240	Pyrimidine metabolism	0.00014368
Joint score test	hsa00230	Purine metabolism	0.000490871
Joint score test	hsa00071	Fatty acid degradation	0.003921085

Table S4: Enriched KEGG pathways in TBT and our Joint Test model

8 JAGUAR

For a given gene-SNP pair, JAGUAR [10] models gene expression across tissues using a linear mixed model in which both fixed and random effects are used to capture the effect of a variant on gene expression. We begin with a linear mixed effects model that models expression patterns across tissues as a function of genotype, i.e.,

$$Y = J\alpha + G\beta + Au + Bv + \xi \tag{13}$$

where Y is a nt-dimensional vector of expression levels in t tissues and n individuals, α is a vector of tissue-specific intercepts, G is a nt-dimensional vector of genotypes, β is a fixed effect of genotype across tissue, $u \sim N\left(0, \tau AA^T\right)$ is a vector of subject-specific random effect, $v \sim N\left(0, \gamma BB^T\right)$ is a vector of tissue-specific random effects, and $\xi \sim N\left(0, \epsilon I_{nt}\right)$. The matrices J, A and B are design matrices with B being a function of genotype. J is $nt \times t$ dimensional matrix denoting the design matrix for the tissue-specific intercepts. A is $nt \times nt$ design matrix for the subject-specific intercepts. B is a $nt \times t$ design matrix of stacked genotypes. The parameters of interest are β and γ; α, τ and ϵ are nuisance parameters.

We test the null hypothesis that $H_0 : \beta = \gamma = 0$, i.e. the variant does not affect gene expression across any of the tissues. To do so, we compute the efficient scores for β and γ by projecting off components correlated with the nuisance parameters.

The efficient scores evaluated under the null are given by –

$$U_\beta = \left(G - \bar{G}\right)^T \hat{\Sigma}_n^{-1} \left(Y - J\hat{\alpha}\right) \tag{14}$$

and

$$U_\gamma = \frac{1}{2} \left(Y - J\hat{\alpha}\right)^T \hat{\Sigma}_n^{-1} BB^T \hat{\Sigma}_n^{-1} \left(Y - J\hat{\alpha}\right) \tag{15}$$

where $\hat{\Sigma} = \hat{\tau}AA^T + \hat{\epsilon}I$ and $\hat{\tau}$ along with $\hat{\epsilon}$ are the maximum likelihood estimators of τ and ϵ under the null.

Following Huang et al [11], we propose a weighted sum of U_β and U_γ to arrive at our joint score test statistic as described by U_ψ. Since U_β is linear in Y while U_γ is quadratic, we propose the following rule to combine them –

$$\begin{aligned} U_\psi &\equiv a_\beta U_\beta^2 + a_\gamma U_\gamma \\ &= \left(Y - J\hat{\alpha}\right)^T \hat{\Sigma}_n^{-1} \left[a_\beta \left(G - \bar{G}\right)\left(G - \bar{G}\right)^T + a_\gamma \left(\frac{1}{2}BB^T\right)\right] \hat{\Sigma}_n^{-1} \left(Y - J\hat{\alpha}\right), \end{aligned} \tag{16}$$

where a_β and a_γ are scalar constants chosen to minimize the variance of U_ψ. Under the null, U_ψ is distributed as a mixture of chi-square random variables. We use Satterthwaite method [1] to approximate the p values from a scaled χ^2 distribution by matching the first two moments as $U_\psi \sim \kappa\chi^2_\nu$ where $\kappa = \frac{2Var(U_\psi)}{E[U_\psi]}$ and $\nu = \frac{2E[U_\psi]^2}{Var(U_\psi)}$.

9 A potential strategy to combine two models to maximize eQTL discovery

In the absence of any tissue-specific methylation effect, our method is underpowered to map eQTLs. One potential way to overcome such situations would be to run an omnibus test that identifies strongest evidence between JAGUAR and our joint test model. Specifically, we calculate the p value under each model, and then compute the minimum of the two p values and compare the observed minimum p value to its null distribution.

DISCLAIMER: We haven't tested this strategy using Monte Carlo simulations. This is a theoretical proposition that could well be wrong.

9.1 Testing the combined effect of methylation and genotype on multi-tissue eQTL detection

In the presence of both methylation and genotypic effect, for a given combination of mRNA, CpG and SNP

$$Y_{MG} = J\alpha + G\beta + M\lambda + MG\phi + Au + Bv + Cw + Dx + \Xi \qquad \Xi \sim (0, \epsilon_{MG}) \tag{17}$$

$u \sim \left(0, \tau AA^T\right)$, $v \sim \left(0, \gamma BB^T\right)$, $w \sim \left(0, \delta CC^T\right)$, $x \sim \left(0, \theta DD^T\right)$. We are interested in the total effect of SNP on gene expression via the null hypothesis

$$H_0 : \beta = \phi = \gamma = \delta = 0$$

The score test statistic is

$$U_{MG} = \left(Y - J\hat{\alpha} - M\hat{\lambda}\right)^T \Sigma_{MG}^{-1} \left[a_\beta \left(G - \bar{G}\right)^T \left(G - \bar{G}\right) + a_\phi \left(MG - \overline{MG}\right)^T \left(MG - \overline{MG}\right) + a_\gamma \frac{1}{2} BB^T + a_\delta \frac{1}{2} CC^T\right] \Sigma_{MG}^{-1} \left(Y - J\hat{\alpha} - M\hat{\lambda}\right) \tag{18}$$

where $\Sigma_{MG}^{-1} = \left(\epsilon I + \tau AA^T + \theta DD^T\right)$

9.2 Testing the effect of genotype on multi-tissue eQTL detection

In the absence of any methylation effect, the model in equation 1 becomes

$$Y_G = J\alpha + G\beta + Av + Bv + \xi \qquad \xi \sim (0, \epsilon_G) \tag{19}$$

$u \sim \left(0, \tau AA^T\right)$, $v \sim \left(0, \gamma BB^T\right)$.

This is essentially JAGUAR. The score test statistic in this case is

$$U_G = (Y - J\hat{\alpha})^T \Sigma_G^{-1} \left[a_\beta \left(G - \bar{G}\right)^T \left(G - \bar{G}\right) + a_\gamma \frac{1}{2} BB^T\right] \Sigma_G^{-1} (Y - J\hat{\alpha}) \tag{20}$$

where $\Sigma_G^{-1} = \left(\epsilon I + \tau AA^T\right)$

Both U_{MG} and U_G follow a mixture χ^2 distribution.

9.3 Omnibus test – a potential strategy

U_{MG} is underpowered in the absence of any methylation effect while U_G is underpowered in the presence of a methylation effect.

- I propose an omnibus test that identifies the strongest evidence among the above two models.
- Specifically, we calculate the p value under both the models, compute the minimum p value and compare the observed minimum p value to its null distribution. We can accomplish this using 1) permutation-resampling method or 2) resampling via perturbation.
- Let $P_{MG} = \mathcal{S}_{\mathcal{MG}}(U_{MG})$ and $P_G = \mathcal{S}_{\mathcal{G}}(U_G)$ be the p values calculated under the two models, where $\mathcal{S}_{MG}(q) = \text{pr}\left\{\widehat{U}^b_{MG} > q; \quad b = 1, \ldots, B\right\}$ and $\mathcal{S}_G(q) = \text{pr}\left\{\widehat{U}^b_G > q; \quad b = 1, \ldots, B\right\}$. $\widehat{U}$ is the permuted or perturbed statistic. B is total number of perturbations or permutations. The null distribution of the minimum p value, $P_{min} = \min(P_{MG}, P_G)$, can be approximated by $\widehat{P}^b_{min} = \min\left(\mathcal{S}_{\mathcal{MG}}\left(\widehat{U}_{MG}\right)^b, \mathcal{S}_{\mathcal{G}}\left(\widehat{U}_G\right)^b\right)$ for all $b = 1, \ldots, B$. Hence, the p value of the omnibus test can be calculated by comparing the observed minimum p value P_{min} with its empirical null distribution $\left\{\widehat{P}^b_{min}\right\}$.
- The permutation p values under each model can be generated by permuting genotypes over all the samples and keeping the permuted order of the genotypes the same for both the models.

10 Reproducibility of the analysis

All the scripts and the accompanied documentation for reproducing our analyses are located at `https://github.com/cramanuj/Epigen_Rcodes`.

References

[1] Satterthwaite F: **An approximate distribution of estimates of variance components**. *Biometrics Bulletin* 1946, **2**(6):110–114.

[2] Bates D, Maechler M, Bolker B, Walker S: *lme4: Linear mixed-effects models using Eigen and S4* 2014, [`http://CRAN.R-project.org/package=lme4`]. [R package version 1.1-7].

[3] Ihaka R, Gentleman R: **A language for data analysis and graphics**. *Journal of Computational and Graphical Statistics* 1996, **5**(3):299–314.

[4] Gibbs J, van der Brug M, Hernandez D, Traynor B, Nalls M, Lai SL, Arepally S, Dillman A, Rafferty I, Troncoso J, Johnson R, Zielke H, Ferrucci L, Longo D, Cookson M, Singleton A: **Abundant quantitative trait loci exist for DNA methylation and gene expression in human brain**. *Plos Genet* 2010, **6**(5).

[5] Purcell S, Neale B, Todd-Brown K, et al: **PLINK: a tool-set for whole-genome association and population-based linkage analyses**. *American Journal of Human Genetics* 2007, **81**(3).

[6] Schmid R, Baum P, Ittrich C, Fundel-Clemens K, Huber W, Brors B, Eils R, Weith A, Mennerich D, Quast K: **Comparison of normalization methods of Illumina BeadChip HumanHT-12 v3**. *BMC Genomics* 2010, **11**.

[7] Leek J, Storey J: **Capturing heterogeneity in gene expression studies by surrogate variable analysis.** *PLoS Genetics* 2007, **3**(9):1724–35.

[8] Fu J, Wolfs M, Deelen P, Westra H, et al: **Unraveling the regulatory mechanisms underlying tissue-dependent genetic variation of gene expression**. *PLoS Genetics* 2012, **8**.

[9] Du P, Zhang X, Huang C, Jafari N, Kibbe W, Hou L, Lin S: **Comparison of Beta-value and M-value methods for quantifying methylation levels by microarray analysis**. *BMC Bioinformatics* 2010, **11**.

[10] Acharya C, McCarthy J, Owzar K, Allen A: **Exploiting expression patterns across multiple tissues to map expression quantitative trait loci**. [Manuscript submitted].

[11] Huang Y, VanderWeele T, Lin X: **Joint analysis of snp and gene expression data in genetic association studies of complex diseases**. *Annals of Applied Statistics* 2014, **8**:352–376.
